# Mortality, morbidity & clinical outcome with different types of vasopressors in out of hospital cardiac arrest patients- a systematic review and meta-analysis

**DOI:** 10.1186/s12872-024-03962-4

**Published:** 2024-05-30

**Authors:** Subhash Chander, Om Parkash, Sindhu Luhana, Abhi Chand Lohana, Fnu Sadarat, Fnu Sapna, Fnu Raja, Zubair Rahaman, Yaqub Nadeem Mohammed, Sheena Shiwlani, NFN Kiran, Hong Yu Wang, Sam Tan, Roopa Kumari

**Affiliations:** 1 Department of Medicine, Icahn School of Medicine at Mount Sinai, New York, NY USA; 2 Department of Medicine, Montefiore Medical Center, Bronx, NY USA; 3 Department of Medicine, AGA khan University Hospital, Karachi, Pakistan; 4 Department of Medicine, Western Michigan University, Kalamazoo, WV USA; 5 Department of Medicine, University at Buffalo, Buffalo, NY USA; 6 Department of Pathology, Montefiore Medical Center, Bronx, NY USA; 7 Department of Pathology, MetroHealth Hospital, Cleveland, OH USA; 8 Department of Pathology, Northwell Health Hospital, New York, NY USA; 9 Department of Pathology, Icahn School of Medicine, Mount Sinai, New York, NY USA

**Keywords:** Cardiac arrest, Ionotropic, ROSC, Vasopressors, Cardiopulmonary resuscitation, Return of spontaneous circulation

## Abstract

**Background & objective:**

Despite their continued use, the effectiveness and safety of vasopressors in post-cardiac arrest patients remain controversial. This study examined the efficacy of various vasopressors in cardiac arrest patients in terms of clinical, morbidity, and mortality outcomes.

**Methods:**

A comprehensive literature search was performed using online databases (MeSH terms: MEDLINE (Ovid), CENTRAL (Cochrane Library), Embase (Ovid), CINAHL, Scopus, and Google Scholar) from 1997 to 2023 for relevant English language studies. The primary outcomes of interest for this study included short-term survival leading to death, return of spontaneous circulation (ROSC), survival to hospital discharge, neurological outcomes, survival to hospital admission, myocardial infarction, and incidence of arrhythmias.

**Results:**

In this meta-analysis, 26 studies, including 16 RCTs and ten non-RCTs, were evaluated. The focus was on the efficacy of epinephrine, vasopressin, methylprednisolone, dopamine, and their combinations in medical emergencies. Epinephrine treatment was associated with better odds of survival to hospital discharge (OR = 1.52, 95%CI [1.20, 1.94]; *p* < 0.001) and achieving ROSC (OR = 3.60, 95% CI [3.45, 3.76], *P* < 0.00001)) over placebo but not in other outcomes of interest such as short-term survival/ death at 28–30 days, survival to hospital admission, or neurological function. In addition, our analysis indicates non-superiority of vasopressin or epinephrine vasopressin-plus-epinephrine therapy over epinephrine monotherapy except for survival to hospital admission where the combinatorial therapy was associated with better outcome (0.76, 95%CI [0.64, 0.92]; *p* = 0.004). Similarly, we noted the non-superiority of vasopressin-plus-methylprednisolone versus placebo. Finally, while higher odds of survival to hospital discharge (OR = 3.35, 95%CI [1.81, 6.2]; *p* < 0.001) and ROSC (OR = 2.87, 95%CI [1.97, 4.19]; *p* < 0.001) favoring placebo over VSE therapy were observed, the risk of lethal arrhythmia was not statistically significant. There was insufficient literature to assess the effects of dopamine versus other treatment modalities meta-analytically.

**Conclusion:**

This meta-analysis indicated that only epinephrine yielded superior outcomes among vasopressors than placebo, albeit limited to survival to hospital discharge and ROSC. Additionally, we demonstrate the non-superiority of vasopressin over epinephrine, although vasopressin could not be compared to placebo due to the paucity of data. The addition of vasopressin to epinephrine treatment only improved survival to hospital admission.

## Introduction

### Study background

Cardiac arrest, also known as cardiopulmonary arrest, refers to the spontaneous loss of blood flow resulting from the inability of the heart to pump blood sustainably to the brain and other vital organs [1]. Cardiac arrest is mainly characterized by dyspnea, hypoxemia, and loss of consciousness [2], which double as signs and symptoms and are often preceded by weakness, chest pain, palpitation, and fluttering.

Cardiac arrest is a major contributor to mortality and morbidity worldwide. In the United States alone, the annual incidence of cardiac arrest is estimated to 295 000, with patients reporting very low survival rates [3]. In most circumstances, advanced cardiac life support guidelines recommend the initiation of an adult basic life support algorithm or defibrillation coupled with enhanced cardiopulmonary resuscitation (CRP) as a fundamental step in the successful management of cardiopulmonary arrest. The main aim of resuscitation is to restart the heart, a condition described as achieving return of spontaneous circulation (ROSC) [4]. In addition to defibrillation and initiation of cardiopulmonary resuscitation, vasopressor therapy has been widely used in the treatment of cardiac arrest.

## Description of intervention (Inotropes)

Pharmacological therapy using vasopressor agents has been the mainstay of early resuscitation, especially for patients with cardiac arrest. Pharmacotherapy usually includes the administration of epinephrine, which is considered the first-line treatment, within 3–5 min intraosseous or intravenously [5]. For instance, 0.5 mg to more than 10 mg epinephrine has been administered through intravenous, intracardiac, endobronchial, and intraosseous routes [6]. Epinephrine is an active sympathomimetic hormone that stimulates the alpha- (α) and beta (β)-adrenergic systems during cardiopulmonary resuscitation [7], thus increasing the probability of achieving return of spontaneous circulation (ROSC). According to previous studies, stimulation of the alpha (α) receptor on the vascular smooth muscles leads to vasoconstriction, which increases aortic diastolic and perfusion pressure to optimize the probability of attaining ROSC [8]. Vasopressin has also been considered as an adjunct or alternative to epinephrine, as it has emerged as superior to epinephrine [9, 10], but this has been equivocal in recent studies.

### Significance of the study

Despite the eminent success of pharmacological therapy through the use of vasopressor agents in managing cardiac arrest, including the management of post-cardiac arrest ROSC, the evidence of their effectiveness is still questionable, as various studies present varied positions. A previous prospective observational study evaluating the effect of vasopressors for cardiac arrest based on the incremental effect on survival rate after out-of-hospital cardiac arrest reported no improvement in survival (odds ratio (OR) 1.1, 95% confidence interval (CI) 0.8 to 1.5), which is attributed to the uncertainty regarding the role of vasopressors in cardiac arrest [11]. Another large prospective study with 417,188 participants with out-of-hospital cardiac arrest showed that although epinephrine improved the rates of ROSC (adjusted OR (aOR) 2.51, 95% CI 2.24 to 2.80), fewer participants survived to or were alive at 30 days (aOR 0.54, 95% CI 0.43 to 0.68) with worse neurological outcomes (aOR 0.21, 95% CI 0.10 to 0.44) raising concerns about epinephrine use. Another recent systematic review of randomized controlled trials by Holmberg et al. [12] also failed to show any significant benefit of vasopressin monotherapy or as an adjunct to epinephrine over epinephrine therapy in enhancing the survival rates of post-cardiac arrest patients. Nonetheless, despite the superiority of epinephrine in improving ROSC rates, studies indicate that it results in no improvement in survival rates, hospital discharge, or favorable neurologic outcomes [13].

Against the above background, a consensus on the effectiveness of vasopressors has yet to be reached. Thus, the present study sought to present a comprehensive review of the literature examining the effectiveness of vasopressors during resuscitation of adults and children in post-cardiac arrest. This study considered a pool of evidence from randomized controlled trials, retrospective studies, prospective studies, or others that were relevant to the most commonly used vasopressors (epinephrine, vasopressin, dopamine, norepinephrine, vasopressin-steroids-epinephrine (VSE)), and also investigate the effects of these vasopressors as monotherapy or combined therapy.

## Study objective

To determine the effectiveness of vasopressors based on mortality, morbidity, and clinical outcomes associated with vasopressor use in cardiac arrest patients.

## Methodology

### Study design

The current study was based on the Preferred Reporting Items for Systematic Reviews and Meta-Analyses (PRISMA) guidelines [14]. A systematic search was performed from January 1996 to February 2023 to answer the research question on the effectiveness of vasopressors on mortality and clinical outcomes in post-cardiac arrest ROSC patients.

### Eligibility criteria: inclusion and exclusion criteria

#### Types of studies

Randomized controlled, prospective and retrospective cohort, and observational studies that compared vasopressors versus placebo or no vasopressors, vasopressors versus other vasopressors, or combined vasopressor regimens versus other vasopressor monotherapy. Only English-language publications and non-English articles that could be interpreted in English were prioritized. This study excluded conference abstracts and editorials.

#### Type of participants

The study included patients with cardiac arrest of all age groups (children and adults), either during hospitalization (IHCA), after hospitalization (OHCA), or shock patients who were treated for cardiac arrest using vasopressors.

#### Type of intervention

Studies that compared various vasopressors as monotherapy against placebo or other vasopressors.

#### Types of outcomes

The major outcomes assessed included all-cause mortality, ROSC, survival to hospital discharge, neurological outcomes, survival to hospital admission, myocardial infarction, and the incidence of lethal arrhythmias.

### Study search process

A comprehensive electronic search was performed on the following databases from January 1996 to February 2023 using text or MeSH terms: MEDLINE (Ovid), CENTRAL (Cochrane Library), Embase (Ovid), CINAHL, Scopus, and Google Scholar. The following combinations of keywords were used in the search process: epinephrine, dopamine, norepinephrine, steroids, vasopressin, heart arrest, cardiac arrest, post-cardiac arrest, resuscitation, and return of spontaneous circulation. A further check on the reference lists of all included studies and relevant systematic reviews and meta-analyses were manually performed to identify additional studies possibly omitted by the database search process.

### Study selection process

Two reviewers (SC and RK) selected the relevant studies. The reviewers began by independently reading the titles and abstracts of all records obtained during the search process to identify relevant studies suitable for inclusion. In case of doubts about a particular title or abstract of a study, the articles were read in the full text. The full publication of all potential studies was retrieved and electronically stored in EndNote. The reviewers (SC and RK) then determined the eligibility of the retrieved articles independently according to the predefined inclusion and exclusion criteria, excluding all studies that contravened the inclusion threshold. Articles published in English were selected for this review. Any disagreements and conflicting decisions regarding any study were addressed via consensus or discussions with the team’s senior reviewers (SL and FS).

### Data extraction

Data extraction was managed by two reviewers (SC and RK). The relevant information was initially abstracted from a paper, transferred to a piloted Cochrane electronic standardized data extraction form, and entered into the Review Manager software (RevMan version 5.4). All crucial study details, including the clinically relevant outcomes, were recorded. Any differences among the reviewers were addressed by re-examining the abstracted data, with further discussion among the reviewers (SC and RK) and consultation with the senior reviewers (SL and FS). Hence, the following information was sought from the selected studies: study characteristics (author’s last name, publication year, study location/setting, and study design), participant demographics (age, mean age or range, gender, percentage of males and females, sample size (n), details of the interventions (vasopressor drugs), study drug vs. control, intervention period, and outcomes.

### Assessment of risk of bias

The potential risk of bias assessment of the included articles was independently performed by two reviewers (SC and RK) using Cochrane Collaboration’s risk of bias tool. It involved author judgments across a series of bias domains per the Cochrane Handbook. The six domains of bias—selection bias, attrition bias, detection bias, performance bias, reporting bias, and the other sources of bias—were evaluated and summarized using Review Manager software as either “low-risk,” “unclear-risk,” or “high-risk.” The studies that showed four low risks in all the domains were considered to have high quality, articles that showed one criterion with high risk or three criteria as unclear were considered to have fair quality, and the studies were categorized as poor quality if they had more than two criteria items with high risk or more than three unclear risks of bias in its domains.

### Statistical analysis

A meta-analysis of the prognostic indexes, such as short-term survival (at 30 days)/ death, survival to hospital discharge, survival to hospital admission, ROSC, and neurologic performance, was conducted using Review Manager (RevMan) software version 5.4 (RevMan 5; The Nordic Cochrane Center, The Cochrane Collaboration, Copenhagen, Denmark). ORs with corresponding 95% CI were extracted from the selected studies to calculate the pooled statistical analysis and determine the association between the vasopressors and potential clinical and mortality indexes or outcomes [15]. Cochrane’s Q test and Higgins I^2^ statistic were used to examine heterogeneity among the included articles. A *p* < 0.05 for the Q-test or I^2^ > 50% for the I^2^ test suggested significant heterogeneity [16] and a random-effect model DerSimonian–Laird method) was applied. Otherwise, the fixed-effects model (Mantel–Haenszel method) was considered. The potential publication bias of the articles was assessed using Begg’s funnel plot asymmetry tests and visual funnel plot diagrams [17, 18].

### Subgroup and sensitivity analysis

If enough data is retrievable, we plan to perform a subgroup and sensitivity analysis where applicable to provide certainty of evidence in case of unusually high heterogeneity. The subgroup and sensitivity analysis will be done on primary outcomes based on different study designs (i.e. RCTs vs. non-RCTs), different study settings, types of initial rhythm used (ventricular fibrillation, external defibrillator use, chest compression), and other quality of studies (low risk of bias, high risk of bias).

## Results

### Study selection and search results

**Fig. 1:**
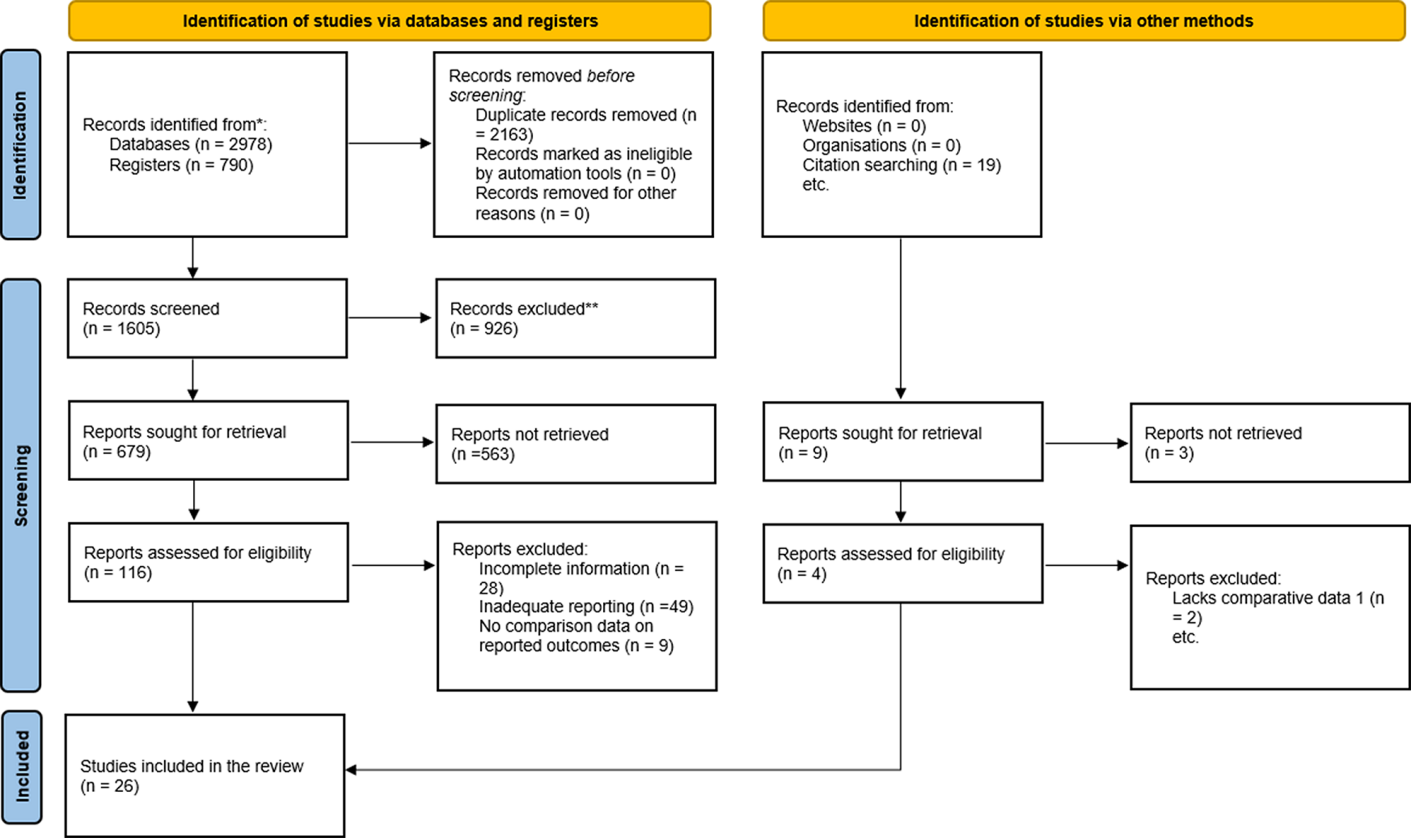
PRISMA flow diagram

An extensive literature search was conducted using databases such as MEDLINE, CENTRAL, Embase, CINAHL, Scopus, and Google Scholar, resulting in 3,768 potential articles. After manually searching reference lists, nine additional studies were found. The reviewers removed 2,163 duplicates, leaving 1,605 articles. After screening for relevance, 116 articles underwent full-text analysis, with 94 excluded. The final review included 22 studies from the database search and four from manual searching, totaling 26 articles that fulfilled the inclusion criteria (Fig. 1).

### Characteristics of included studies

In this meta-analysis, 26 studies, including 16 RCTs [19–34] and 10 non-randomized cohort studies [35–44]) that met the predefined criteria considered. The randomized controlled studies comprised 6 single-centre RCTs [19–21, 23, 31, 32], eight 8 multi-centre RCTs, and two 2 single-centre non-RCTs [39, 43]. Participants were adults aged 30–80, with two exceptions involving ages 15–94 [38] and 16–21 [26]. Males were predominant in all study populations. The sample size totaled 459,708 participants, with individual study sizes ranging from 40 to 417,188 [36]. Studies were globally distributed and published between 1997 and 2021. Full characteristics of the included studies can be found in Table 1.

**Table 1:** Characteristics & summary of included studies

First author (Year)	Location	Design	Participant characteristics/ demographics		Intervention vasopressors		Intervention period	Outcomes	Conclusion
			**Size (N)**	**Age; mean, ranges**	**Study grp**	**Control grp**			
Jacobs et al. [19] (2011)	Australia	RCT	601	~ 64 yrs.	Epinephrine = 272 patients	Placebo 262 patients	NR	-Survival to hospital discharge -ROSC -Cerebral/neurologic performance	Epinephrine demonstrated increase (OR 3.4; 95% CI 2.0-5.6) but not survival to hospital discharge (OR 2.2; 95% Cl 0.7–6.3). Two patients in epinephrine group had unfavorable neurological outcomes.
Nordseth et al. [20] (2012)	Norway	RCT	174	adult (> 18 years)	Epinephrine = 101	Placebo = 73	May 2003 and April 2008	-ROSC	Epinephrine increases the rate of ROSC.
Olasveengen et al. [35] (2012)	Norway	Cohort	848	adult (> 18 years)	Epinephrine = 367	Placebo = 481	May 1st 2003 and April 28th, 2008	-Short-term survival/ death -Survival to hospital discharge, -Neurologic outcomes	Epinephrine is associated with improved short-term survival but decreased survival to hospital discharge and survival with favorable neurologic outcomes. -OR 2.5 (Cl 1.9, 3.4), 0.5 (Cl 0.3, 0.8) and 0.4 (Cl 0.2, 0.7) respectively.
Olasveengen et al. [21] (2009)	Norway	RCT	1,183	64 (18)	418 patients received Epinephrine	433 Placebo	May 1, 2003, and April 28, 2008	-Hospital admission with ROSC -Survival to hospital discharge, -Survival with favorable neurological outcome	Epinephrine patients had higher rates of short-term survival with ROSC. (32% vs. 21%, *p* < 0.001) No improvement in survival to hospital discharge or long-term survival. 10.5% vs. 9.2%, *p* = 0.61) and (10% vs. 8%, *p* = 0.53) respectively
Hagihara et al. [36] (2012)	Japan	Observational Study	417,188	72.38 (15.5)	Epinephrine = 15 030 patients	No-drug (placebo) = 402 158 patients	Jan 1st, 2005-Dec 31, 2008	-ROSC -Survival to hospital discharge -Survival with good neurologic/ cerebral performance	-Positive association with pre-hospital epinephrine and ROSC. (OR 2.36%; 95% Cl, 2.22–2.50; *p* < 0.001) -Negative association with pre-hospital epinephrine with survival to hospital discharge and good neurological outcome.
Hayashi et al. [37] (2012)	Japan	Observational study	3,161	73.3 (15.2)	1,013 received epinephrine	2148 received no drug	January 2007 through December 2009.	-Short-term survival, hospital admission -ROSC -Neurological outcome survival	Early administration of epinephrine improved ROSC (13.4% vs. 29.3%, *p* < 0.001) and neurological outcomes (4.1% vs. 6.1%, *p* = 0.028). No favorable outcome for hospital admission and short-term survival.
Nakahara et al. [38] (2013)	Japan	Cohort study	1,990	15–94 yrs.	Epinephrine (*n* = 2464)	No epinephrine (*n* = 12 479)	January 2007 to December 2010.	-Overall survival, -Neurologic outcomes	Epinephrine administration improved outcomes in patients for overall survival (OR 1.34, 95% CI 1.2 to 1.6). No favorable outcome for neurological intact survival.
Chiang et al. [39] (2015)	Taiwan	Cohort Study	514	~ 48 yrs.	Epinephrine group = 43	No-drug/ placebo grp = 471	June 1, 2010, to May 31, 2013	-ROSC -Survival to discharge	Administration of epinephrine was associated with increased short-term survival.
Perkins et al. [22] (2018)	UK	RCT	8014	69.7 ± 16.6	epinephrine (4015 patients)	saline placebo (3999 patients)	December 2014 through October 2017	-Rate of survival at 30 days -Rate of survival until hospital discharge -Favorable neurologic outcome	Epinephrine resulted in higher 30-day survival 3.2% vs. 2.4% (OR 1.39; 95% CI 1.06, 1.82; *p* = 0.02) but no difference in neurologic outcomes (2.2% vs. 1.9% (OR 1.18; 95% Cl, 0.86, 1.61).
Goto et al. [40] (2013)	Japan	Observational study	15,492	74.0 (± 16.1)	Epinephrine (*n* = 3,136)	Placebo (*n* = 12,356)	January 2009 to December 2010.	-ROSC − 1-month survival − 1-month favorable neurological outcomes	ROSC in non-shockable Epinephrine group was significantly higher than non-epinephrine group (18.7% vs. 3.0%, p0.001). 1-month survival in epinephrine group was significantly higher than non-epinephrine group (3.9% vs. 2.2% *p* < 0.001). No favorable neurological outcome in the Epinephrine group.
Kim et al. [23] (2022)	Korea	RCT	148 adults	77.0 (68.3–83.0)	Vasopressin with epinephrine (*n* = 74)	Placebo with epinephrine (*n* = 74)	August 2017 to August 2021	-ROSC, -Survival to discharge, and neurologic outcomes at discharge.	No significant increase in ROSC in the vasopressin group as compared to placebo (36.5% vs. 32.4%, RR 4.1%; *p* = 0.60). Survival discharge and neurological outcomes did not differ between the groups.
Dumas et al. [41] (2014)	USA	Cohort	1,556	60 ± 16 years	Epinephrine 1,134 patients	No drug/placebo 422	January 2000 to August 2012	-Survival to hospital discharge -Survival with good neurological	Pre-hospital epinephrine use is associated with lower survival chances.
Ong et al. [26] (2012)	Singapore	RCT	727	aged > 16 (aged > 21 for one hospital)	Epinephrine = 353	Vasopressin = 374	9 March 2006 to 19 January 2009	-Survived to hospital discharge -Survived to hospital admission	The combination of vasopressin and epinephrine did not improve long-term survival, but it improved survival at hospital admission. 22.2% vs. 16.7% (*p* = 0.05, RR = 1.43, 95% Cl = 1.02–2.04).
Stiell et al. [27] (2001)	Canada	RCT	200	70 (14)	104 patients received vasopressin	96 received epinephrine	July 3, 1997, to Nov 30, 1998	-Survival to hospital discharge -Survival to 1 h, and neurological function, and myocardial ischemia	There was no survival advantage for vasopressin over epinephrine (12% vs. 14%; *p* = 0.67; 95% Cl − 11.8–7.8%). 1-h survival (39% vs. 35%; *p* = 0·66; Cl 10·9% to 17·0%)
Wenzel et al. [30] (2004)	Austria, Germany, and Switzerland	RCT	1186	66.5 ± 14.4	589 received vasopressin	597 received epinephrine	June 1999 to March 2002	-Survival to hospital admission -Survival to hospital discharge	Effect of vasopressin were similar to epinephrine in managing ventricular fibrillation and pulseless electrical activity, but vasopressin was superior in asystole patients. Hospital admission (29.0% vs. 20.3%; *p* = 0.02) and hospital discharge (4.7% vs. 1.5%, *p* = 0.04). Vasopressin plus epinephrine was more effective than epinephrine alone.
Lindner et al. [42] (1997)	Germany	randomized comparison	40 patients	65 ± 4 years	epinephrine (*n* = 20)	vasopressin (*n* = 20)	July, 1994, to December, 1995	-Hospital admission -Survival for 24 h, -Survival to hospital discharge and neurological outcome	Significantly larger proportion of patients treated with vasopressin than of those treated with epinephrine were resuscitated successfully from out-of-hospital ventricular fibrillation and survived for 24 h.
Gueugniaud et al. [29] (2008)	France	RCT	2894	75.4	r 1 mg of epinephrine and 40 IU of vasopressin (1442)	Epinephrine and saline placebo (1452)	May 1, 2004, through April 30, 2006	-Survival to hospital admission -ROSC -Survival to hospital discharge - Good neurologic recovery -1-year survival	Compared with epinephrine alone, combined vasopressin plus epinephrine does not improve outcome. Hospital admission: (20.7% vs. 21.3%, RR 1.01; 95% CR 0.97 to 1.05). ROSC (28.6% vs. 29.5%; RR 1.01; 95% CI, 1.00 to 1.02) Neurological recovery (37.5% vs. 51.5% RR 1.29; 95% CL 0.81 to 2.06)
Turner et al. [43] (2014)	USA	Observational Study	101	51 (33–65)	vasopressin plus epinephrine = 43	Epinephrine alone = 58	July 2010 to July 2012	-ROSC -Survival to hospital discharge	In combination with epinephrine, vasopressin demonstrated improved ROSC without improving the survival to hospital discharge. (63% vs. 37%, *p* = 0.01).
Guyette et al. [44] (2004)	USA	Observational	298 subjects	65 ± 15	vasopressin plus epinephrine (*n* = 37)	Epinephrine alone (*n* = 231)	March 2002 to March 2003	ROSC	A combination of vasopressin plus epinephrine had more ROSC and hospital arrival than epinephrine alone. (LR: 2.73; 95% Cl, 1.24, 6.03 | LR 3.85; 1.75, 8,65) respectively.
Ducros et al. [28] (2011)	France	RCT	44	56 ± 2	-Vasopressin plus epinephrine (*n* = 14) -Vasopressin plus epinephrine plus nitroglycerin (*n* = 14)	Epinephrine alone (*n* = 16)	August 2001 to August 2004	ROSC	No significant difference with the addition of vasopressin or vasopressin plus nitroglycerin to epinephrine to achieve the ROSC compared to epinephrine alone in in cardiac arrest patients.
Mentzelopoulos et al. [31] (2013)	Greece	RCT	268	63.2 (17.6)	vasopressin-steroids-epinephrine (VSE) combination (*n* = 130)	Epinephrine with Saline (*n* = 138)	September 1, 2008, to October 1, 2010	ROSC, survival to hospital discharge, favorable neurological status, number of organ failure-free days	Combined vasopressin-epinephrine and methylprednisolone compared with epinephrine/saline resulted in improved survival and hospital discharge with favorable neurological status. ROSC 83.9% vs. 65.9%; OR 2.98; 95% Cl 1.39–6.40; *p* = 0.005. Survival to hospital discharge 13.9% vs. 5.1%, OR 3.28; 95% Cl, 1.17–9.20; *p* = 0.o2
Mentzelopoulos et al. [32] (2009)	USA	RCT	100 potentially eligible patients	65.4 (17.6)	Vasopressin plus epinephrine & methylprednisolone (*n* = 48)	Epinephrine (*n* = 52)	June 8, 2006, to March 16, 2007	return of spontaneous circulation, survival to hospital discharge, organ failure-free days	Combined vasopressin-epinephrine and methylprednisolone during resuscitation improved survival in refractory in-hospital cardiac arrest. ROSC- 81% vs. 52%; *p* = 0.003 Improved survival to hospital discharge 19% vs. 4%; *p* = 0.02.
Botnaru et al. [33] (2014)	Canada	RCT	300	N/A	VSE (*n* = 146)	Epinephrine alone (*n* = 154)	Sep 2008 to Oct 2010	-ROSC -Survival to hospital discharge -Neurological outcome at discharge -Steroid SE	VSE was associated with increased survival to hospital discharge and favorable neurologic outcomes compared to Epi.
Andersen et al. [24] (2021)	Denmark	RCT	501	~ 70 years	Vasopressin + methylprednisolone (237)	Epinephrine (*n* = 264)	Oct 15, 2018, to Jan 21, 2021.	ROSC	Vasopressin + methylprednisolone, compared with epinephrine, significantly increased the likelihood of ROSC, but it is uncertain whether there is benefit or harm for long-term survival.
Granfeldt et al. [25] (2022)	Denmark	RCT	501 patients	71 (13) years	Vasopressin plus methylprednisolone (237)	Epinephrine (*n* = 264)	N/A	-Survival, -Survival with favorable neurological outcomes and health-related quality of life	Administration of vasopressin and methylprednisolone, compared with epinephrine, in patients with in-hospital cardiac arrest did not improve long-term outcomes.
De Backer et al. [34] (2010)	Belgium, Austria, and Spain	multi-center, randomized trial	1679 patients	55–76 years	Dopamine (*n* = 858)	Norepinephrine (*n* = 821)	December 19, 2003, and October 6, 2007	-Rate of death at 28 days -Occurrence of adverse events	Dopamine, as compared with norepinephrine, was associated with an increased rate of death at 28 days. (52.5% in the dopamine group and 48.5% in the norepinephrine group; OR 1.17; 95% confidence interval, 0.97 to 1.42; *P* = 0.10) There were more arrhythmic events among the dopamine than the norepinephrine group (207 events [24.1%] vs. 102 events [12.4%].

### Risk of bias assessment

**Fig. 2:**
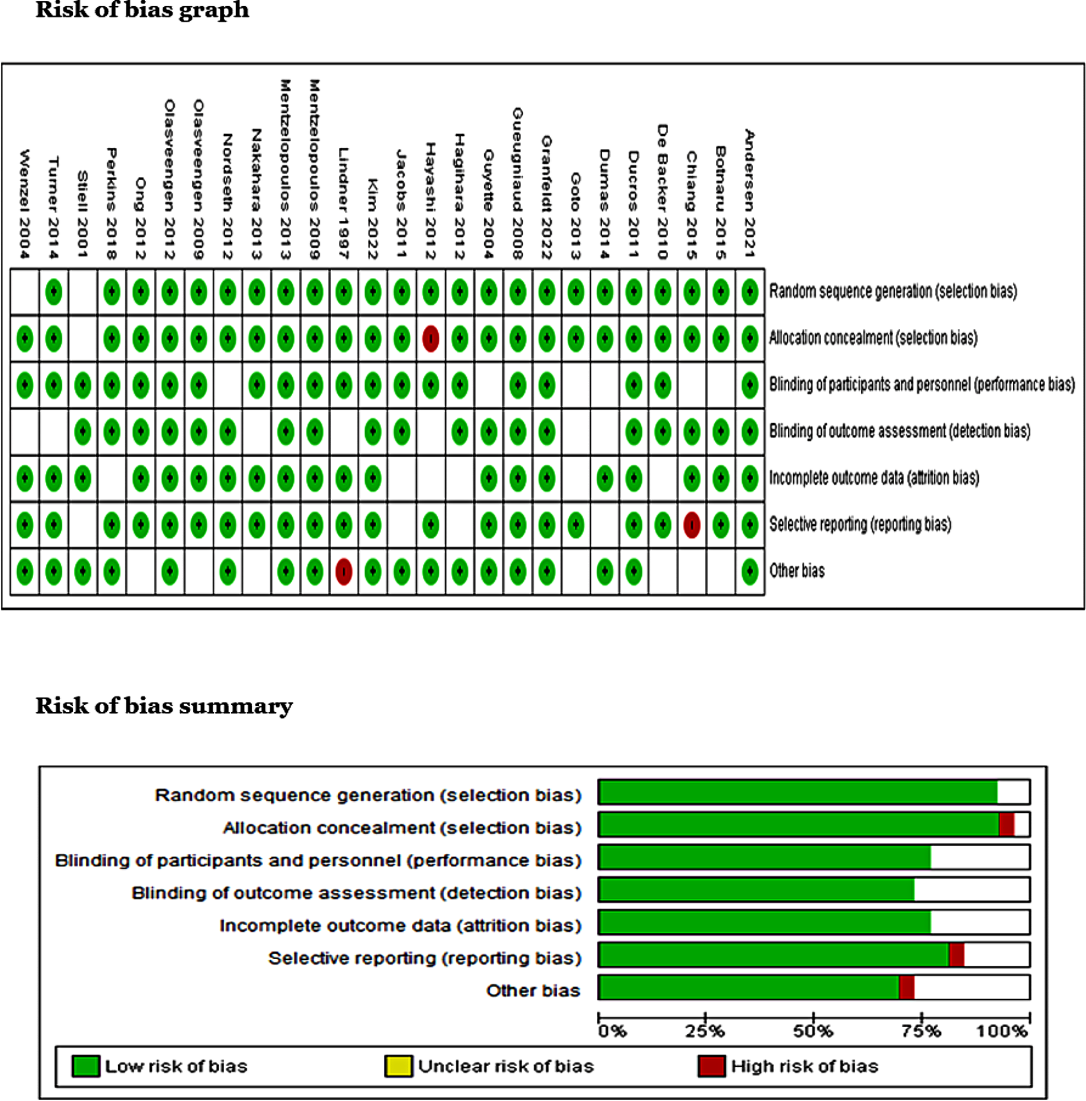
Risk of bias assessment (graph & summary)

The studies included in this review were assessed for their quality and risk of bias using Cochrane Collaboration’s risk of bias tool, as illustrated in (Fig. 2). Most studies were judged to be of high quality based on the risk of the assessment tool. However, five studies were classified as fair quality for the following reasons: in the studies by Stiell et al., Guto et al., and Dumas et al., three elements (i.e., participant blinding, allocation concealment, and selective reporting) were not clearly classified. Chiang et al. and Lindner et al. had high risks for some elements (i.e., allocation concealment and selective reporting).

## Evidence and synthesis for clinical and mortality outcomes

### Epinephrine vs. no drug/placebo

#### Short-term survival/ death at 28–30 days

**Fig. 3:**
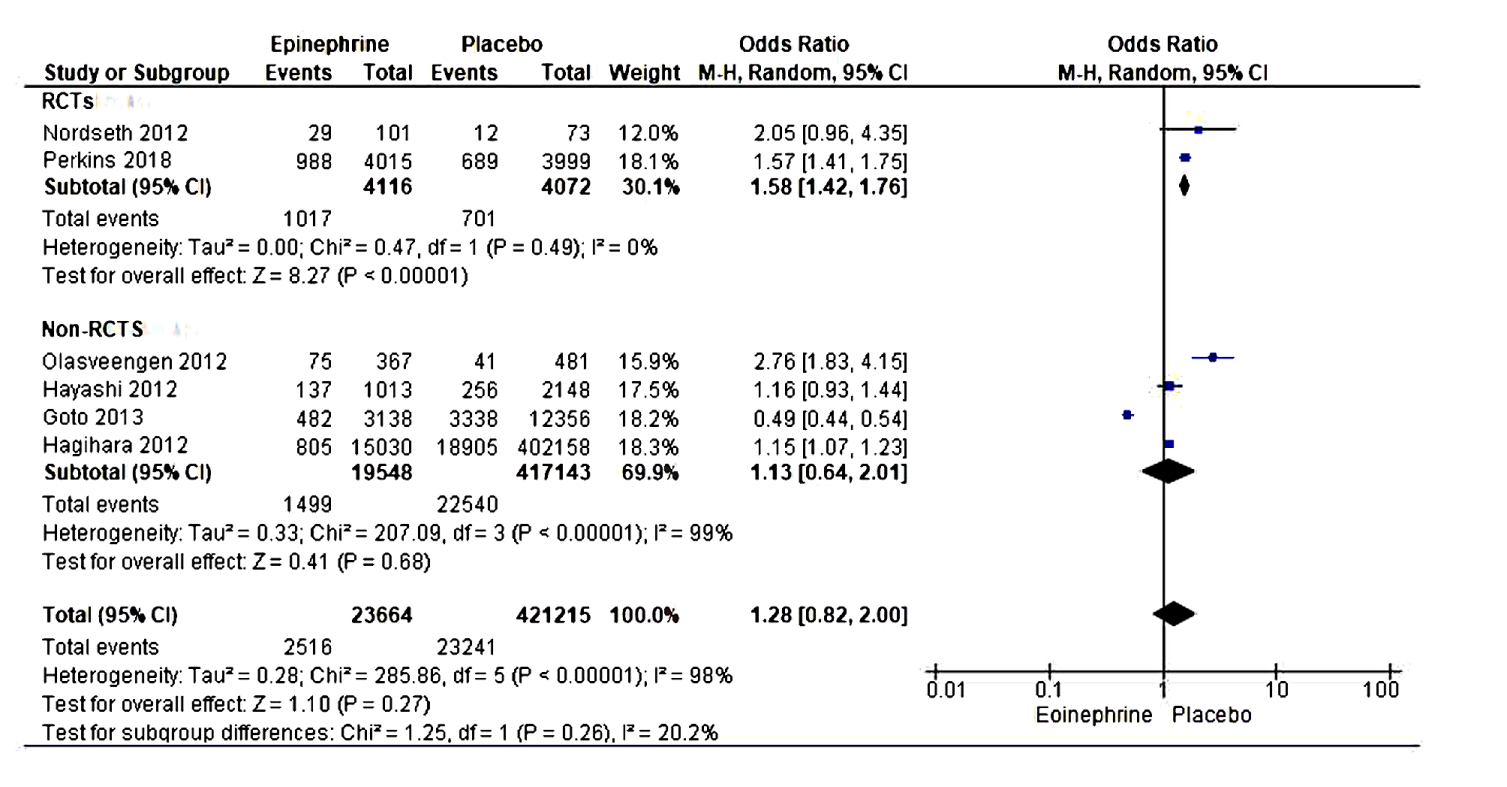
Forest plot of comparison (epinephrine vs placebo): short-term survival/death within 30 days

Six studies with 4,44,879 cardiac arrest patients reported mortality-related short-term survival within 30 days in the epinephrine group compared with the placebo group [20, 22, 35–37, 40]. The meta-analysis showed a significant statistical difference in short-term survival risks or death within 30 days in the placebo than in the epinephrine treatment group. However, the high heterogeneity realized in this analysis rendered this result inconsistent and was not pooled for consideration. A subgroup analysis was performed to assess the potential source of the high heterogeneity. The type of studies included might have been the source of heterogeneity. In the subgroup analysis, two randomized trials were included, and results showed statically significant differences between epinephrine compared to the placebo group on short-term survival within 30 days (OR = 1.58, 95% CI [1.42, 1.76], *P* < 0.00001). No dissimilarities were revealed through the heterogeneity test (Tau² = 0.00; Chi² = 0.47, df = 1 (*P* = 0.49); I² = 0%). The test for subgroup differences revealed low heterogeneity (Chi² = 1.25, df = 1 (*P* = 0.26), I² = 20.2%)(Fig. 3).

#### ROSC

**Fig. 4:**
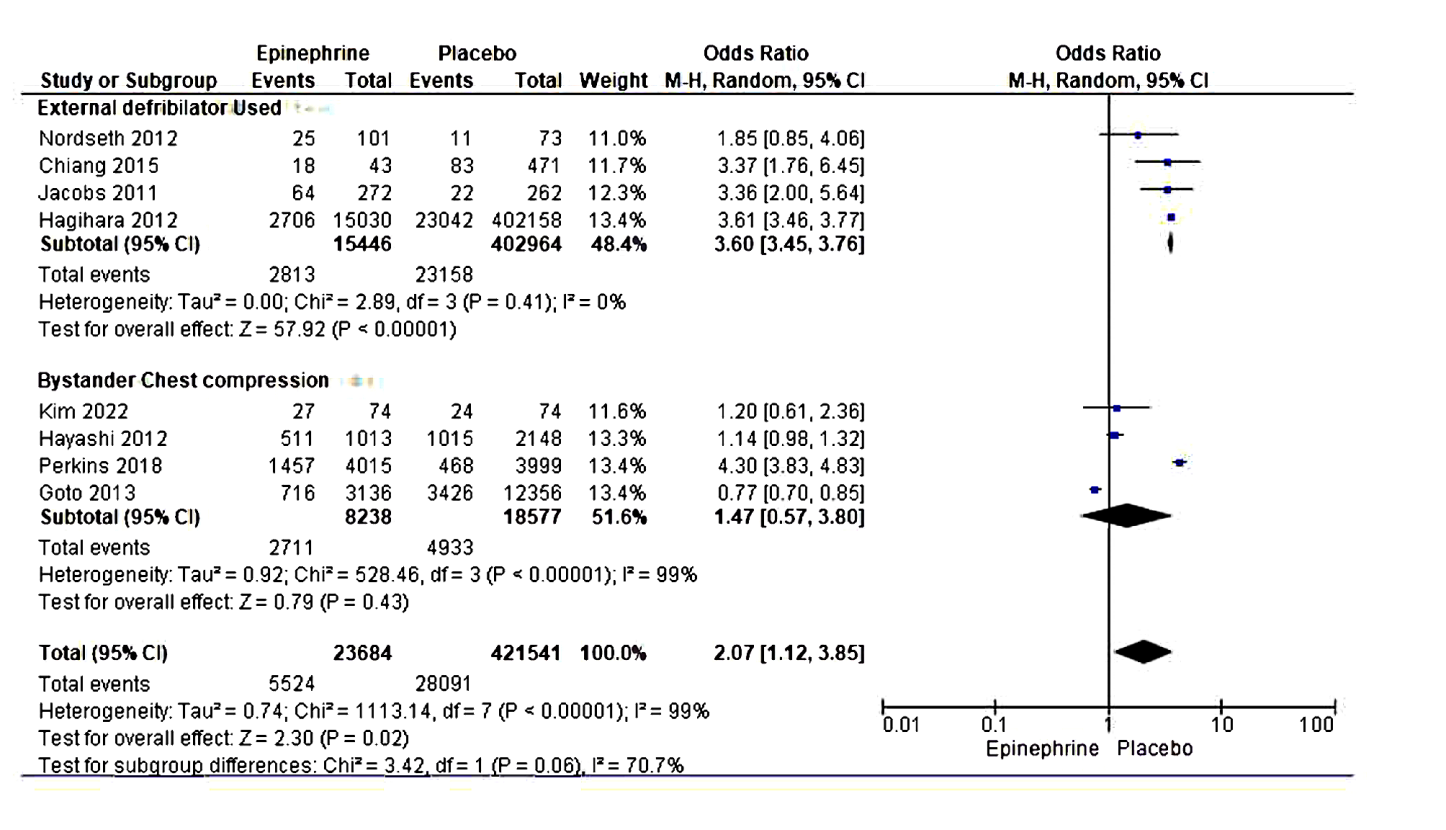
Forest plot of comparison (epinephrine vs placebo): achieving ROSC.

Eight studies with 445,225 participants reported rates of ROSC [19–23, 36, 37, 39, 40]. When all the studies were pooled, the meta-analysis showed a statistically significant difference between the adrenaline and placebo treatments. However, this result could not be pooled for consideration due to the high heterogeneity detected between the included studies. A subgroup analysis using randomized and non-randomized studies independently still exhibited high heterogeneity. A further sub-group analysis was performed to establish the source of high heterogeneity, and studies were categorized into those that deployed ventricular defibrillators or external defibrillators and those using normal chest compression for resuscitation. The results maintained a statistically significant difference between the epinephrine intervention and placebo group (OR = 3.60, 95% CI [3.45, 3.76], *P* < 0.00001), with low dissimilarities between the studies ((Tau² = 0.00; Chi² = 2.89, df = 3 (*P* = 0.41); I² = 0%)); (Fig. 4). The test for subgroup differences revealed high, though considerable, heterogeneity (Chi² = 3.42, df = 1 (*P* = 0.06), I² = 70.7%). Through a sensitivity test, the authors established that high variability in data sources, cardiac arrest causes (trauma vs. non-trauma inclusion), varied response time and inclusion of broader age ranges might also account for the high heterogeneity witnessed.

#### Neurological function

**Fig. 5:**
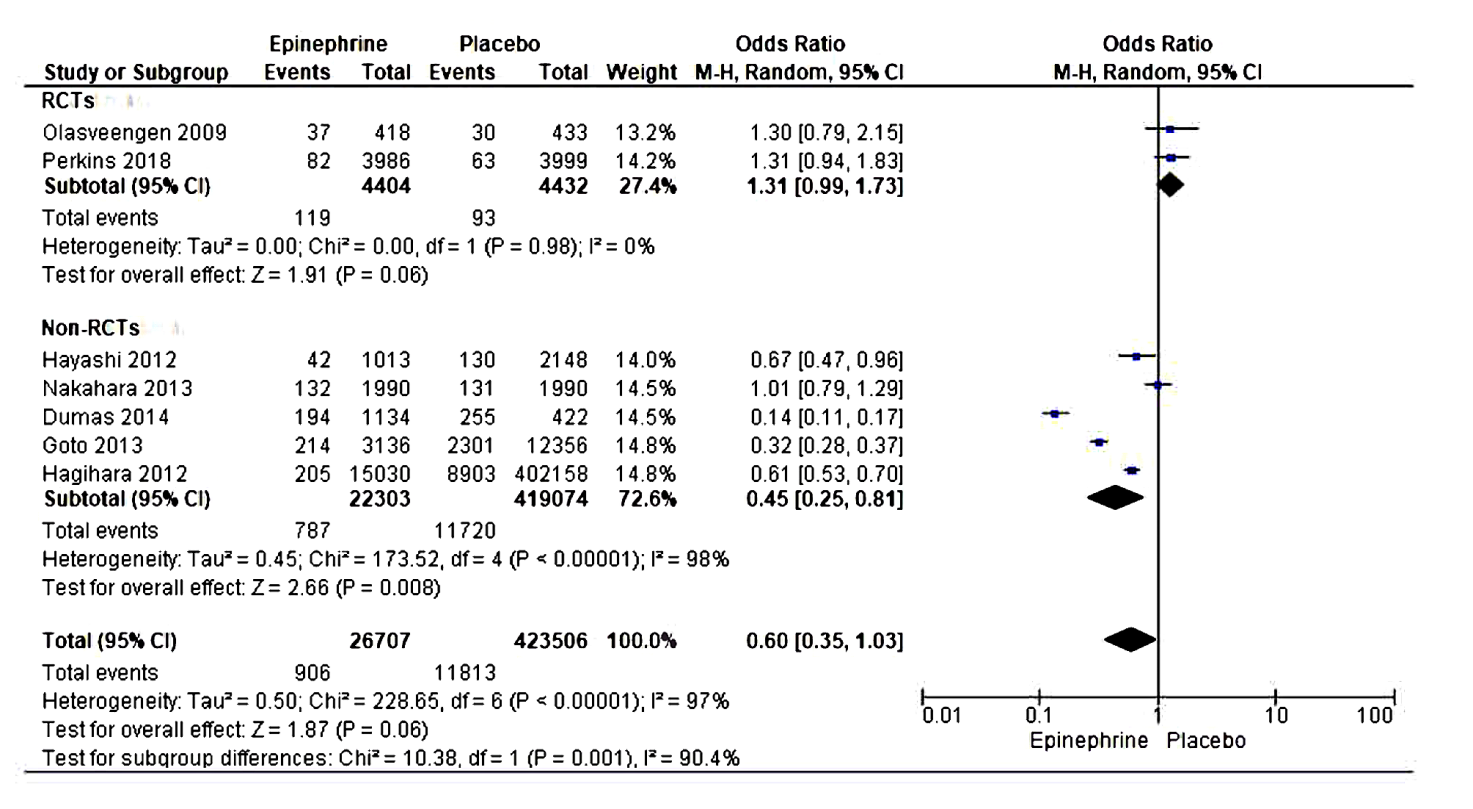
Forest plot of comparison (epinephrine vs placebo): neurologic function

This study included seven high-quality articles with a pooled participant size of 4,50,193 reporting neurological function, comparing epinephrine use versus placebo to enhance neurological performance [21, 22, 36–38, 40, 41]. A random-effect model was used to estimate the effect size between the intervention groups statistically. The meta-analysis showed a statistically non-significant association between epinephrine use and improved neurological function compared to the placebo or no-drug intervention. However, these results could not be pooled due to the significant heterogeneity revealed, showing high dissimilarity between the evaluated studies. The authors conducted a subgroup analysis by independently conducting a meta-analysis for RCTs and then observational non-RCTs. The sub-group analysis revealed that including observational (non-RCT) studies might have been a potential source of high heterogeneity due to bias in participant selection. A meta-analysis including two studies showed a statistically significant difference between the epinephrine and placebo group on the neurological function outcomes (OR = 1.31, 95% CI [0.99, 1.73], *P* = 0.06), suggesting that epinephrine potentially improved neurologic outcomes of cardiac arrest patients. The heterogeneity test revealed no significant variation between the assessed studies (Tau² = 0.00; Chi² = 0.00, df = 1 (*P* = 0.98); I² = 0%).(Fig. 5).

#### Survival to hospital admission

**Fig. 6:**
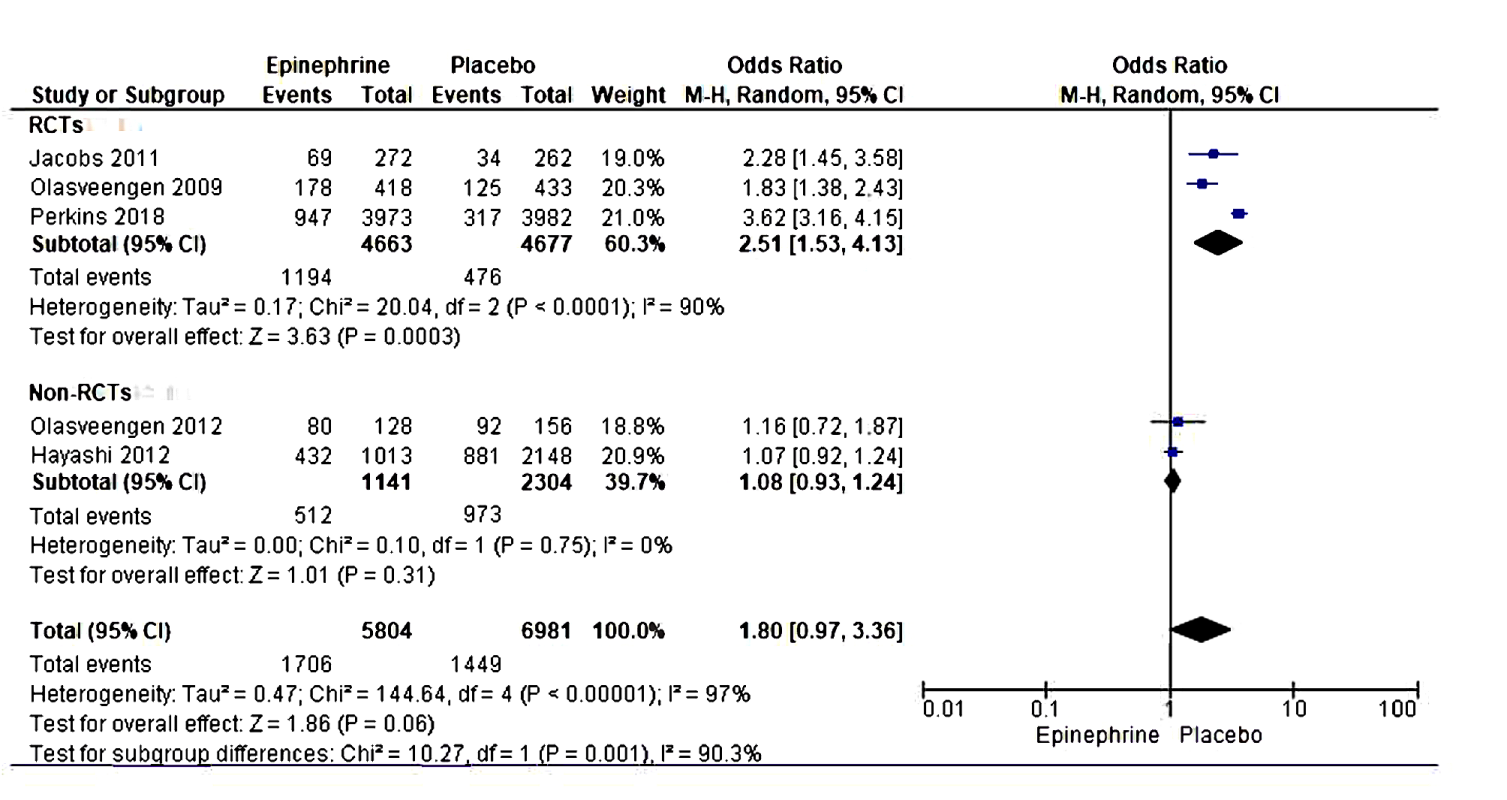
Forest plot of comparison (epinephrine vs placebo): survival to hospital admission

Five studies, including 12,785 patients, reported data on survival-to-hospital admission outcomes in epinephrine versus placebo groups [19, 21, 22, 35, 37]. The meta-analysis results showed a non-significant statistical difference in survival to hospital admission between epinephrine and placebo, suggesting that administering epinephrine slightly enhanced patient survival until the arrival of emergency care or the receiving hospital. However, these results were compromised due to the high heterogeneity between the five studies; hence, they could not be pooled. Further assessment was done to ascertain the source of high heterogeneity. A subgroup analysis using two observational studies ascertained the above findings, with the meta-analysis results showing no difference between the intervention groups (OR = 1.08 [0.93, 1.24], *P* = 0.31), with no inter-study heterogeneity (Tau² = 0.00; Chi² = 0.10, df = 1 (*P* = 0.75); I² = 0%). Further sensitivity analysis in the RCT studies noted that differences in study settings and recruitment periods could also contribute to high heterogeneity within studies (Fig. 6).

#### Survival to hospital discharge

**Fig. 7:**
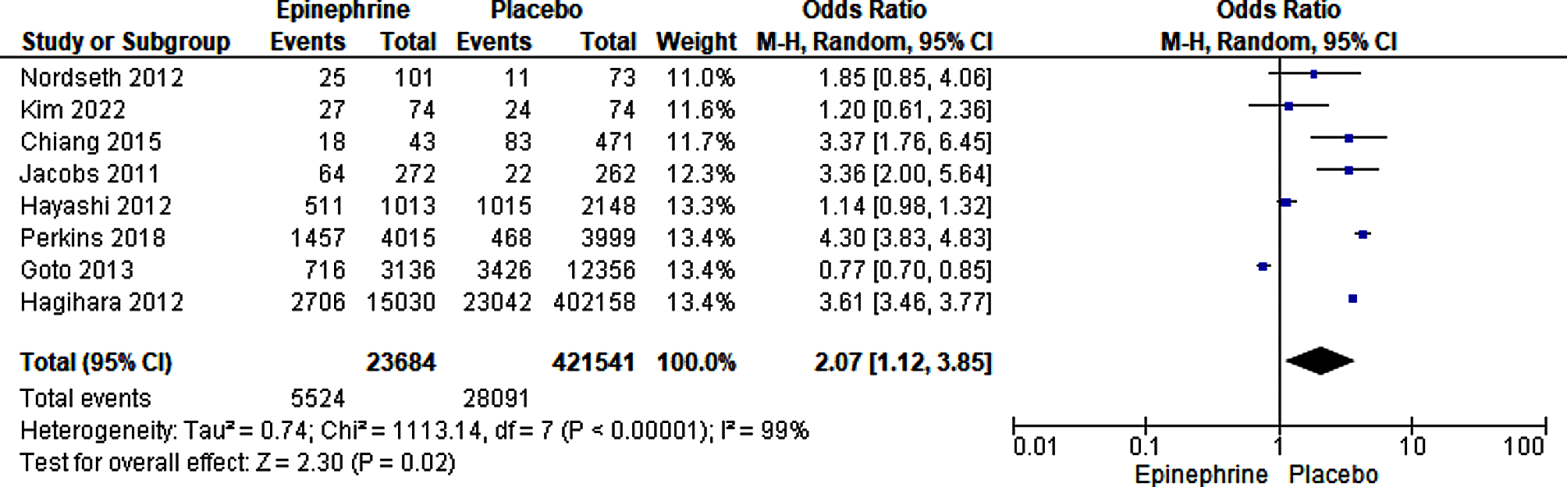
Forest plot of comparison (epinephrine vs placebo): survival to hospital discharge

The study assessed the association of survival to hospital discharge with epinephrine compared with placebo by including six studies with 14,028 participants [19, 21–23, 38, 39]. The inter-study heterogeneity tests showed low heterogeneity (I²=42%, *p* = 0.13). The meta-analysis results using the fixed effect model showed significantly better odds of survival to discharge in the placebo group than in the epinephrine group (OR = 1.52, 95%CI [1.20, 1.94]; *p* < 0.001)(Fig. 7).

### Epinephrine vs. Vasopressin

#### Survival to hospital discharge

**Fig. 8:**
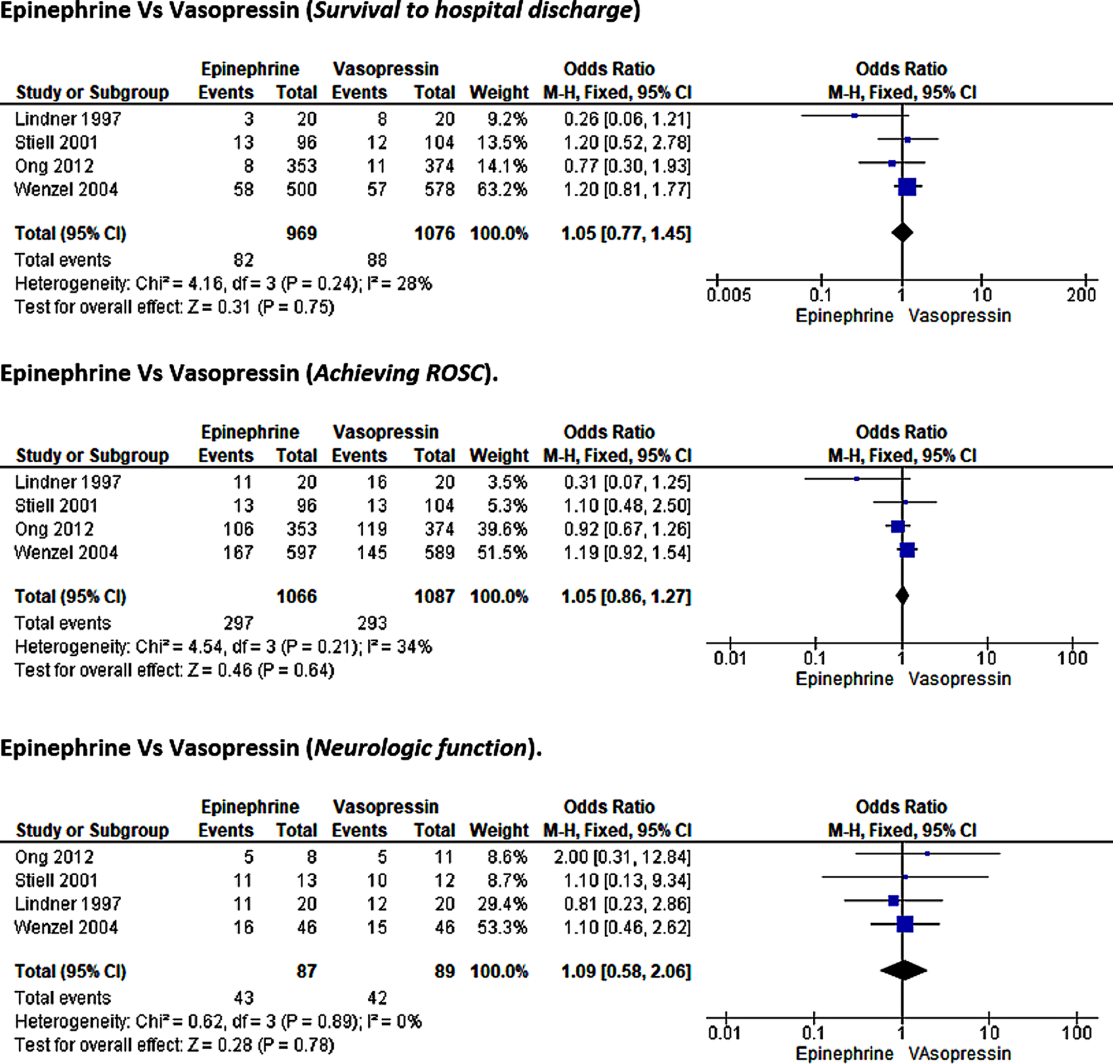
Forest plot of comparison: epinephrine vs vasopressin

Furthermore, the study reported survival to hospital discharge outcomes by comparing the epinephrine and vasopressin treatment arms. The meta-analysis included four RCT studies with 2,045 participants—969 on epinephrine and 1,076 on vasopressin [26, 27, 30, 42]. In the meta-analysis using the fixed model method, the statistical pooled results (OR = 1.05, 95%CI [0.77, 1.45]; *p* = 0.75) demonstrated a non-significant effect of vasopressin compared to the epinephrine group in improving survival to discharge outcomes in post-cardiac arrest ROSC patients. Low heterogeneity was observed in this subgroup analysis (I²=28%, *p* = 0.24)(Fig. 8).

#### ROSC

Four randomized controlled trials, including 2,153 patients, compared the efficacy of epinephrine and vasopressin in achieving ROSC [26, 27, 30, 42]. Based on the pooled statistical results using the fixed-effects model, there was no statistically significant difference between the administration of epinephrine and vasopressin (OR = 1.05, 95%CI [0.86, 1.27]; *p* = 0.64) in cardiac arrest patients with low heterogeneity among the studies (I²=34%, *p* = 0.21)(Fig. 8).

#### Neurologic function

Finally, four studies, including 176 participants, reported on neurologic functions with epinephrine and vasopressin interventions [26, 27, 30, 42]. The heterogeneity test revealed no variation among the assessed studies (I²=0%, *p* = 0.89). In the meta-analysis, the pooled statistical results revealed a non-significant effect of vasopressin compared to the epinephrine group (OR = 1.09, 95%CI [0.58, 2.06]; *p* = 0.78) (Fig. 8).

### Vasopressin + epinephrine vs. epinephrine

#### Survival to hospital discharge

**Fig. 9:**
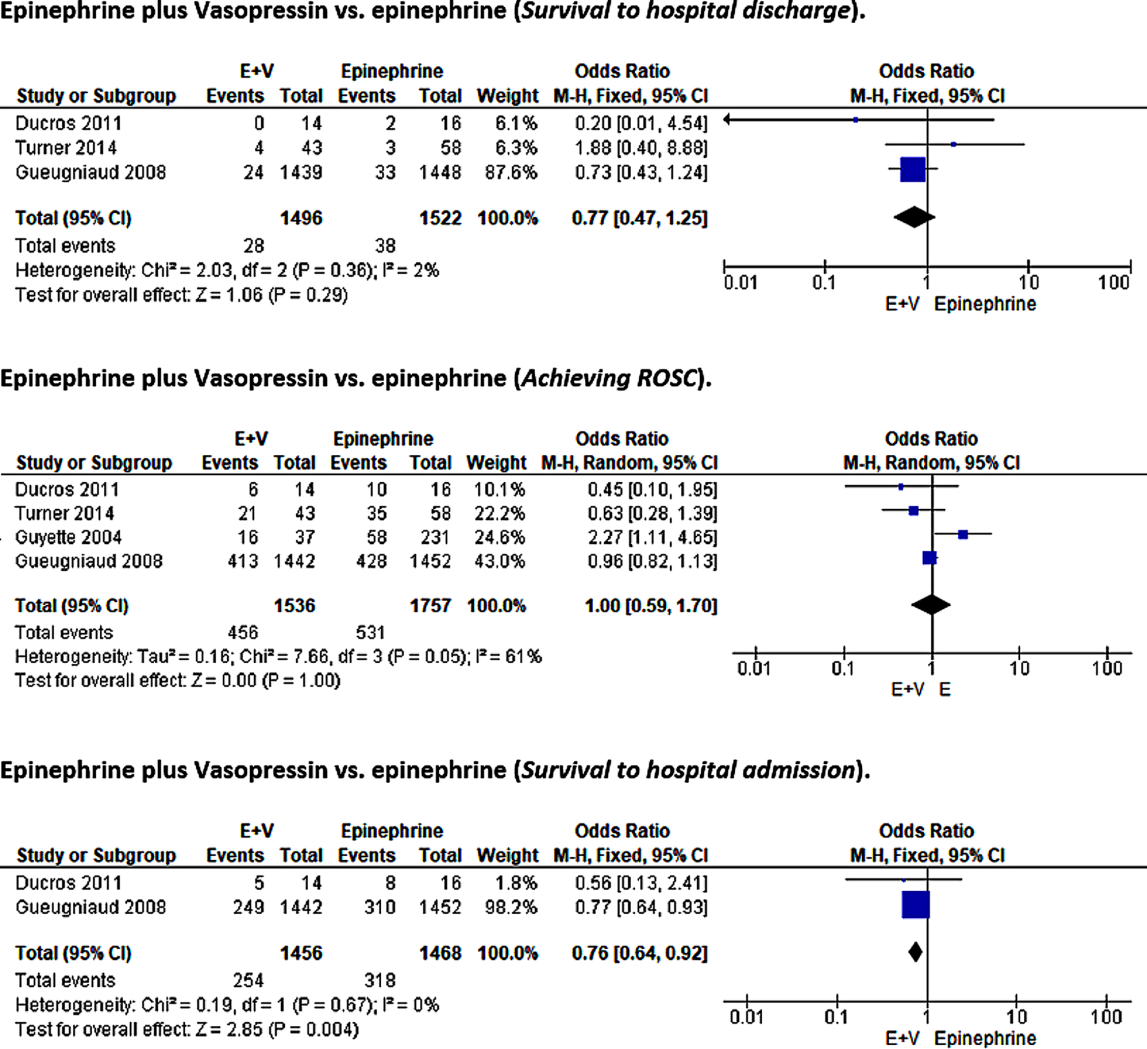
Forest plot comparison: epinephrine plus vasopressin vs. epinephrine

Three included studies with 3,018 participants reported data for comparing the survival to hospital discharge outcomes with combinatorial therapy with vasopressin and epinephrine (VE) therapy against monotherapy with epinephrine [28, 29, 43]. Heterogeneity among the three studies was low (I²=2%, *p* = 0.36), suggesting insignificant variance; hence, a fixed-effects model was adopted for the statistical analysis. The pooled meta-analysis results showed no statistical differences in the risk of survival to hospital discharge with VE or epinephrine monotherapy (OR = 0.77, 95%CI [0.47, 1.25]; *p* = 0.29) in post-cardiac arrest ROSC patients (Fig. 9).

#### ROSC

This study also assessed ROSC in patients treated with VE versus epinephrine. Pooled analysis of four studies with 3,293 participants [28, 29, 43, 44] revealed no difference in hospital discharge outcomes between the two treatment arms (OR = 1.00, 95%CI [0.59, 1.70]; *p* = 1.00). Because inter-study heterogeneity was notable (I²=61%, *p* = 0.05), significant variation was assumed; hence, a random-effects model was used for pooled statistical effects (Fig. 9).

#### Survival to hospital admission

Finally, two studies with 2,924 participants allowed the comparison of survival to hospital admission improvement outcomes [28, 29]. The included studies had no heterogeneity (I²=0%, *p* = 0.67); hence, a fixed-effect model was used for the meta-analysis. The pooled results of the meta-analysis showed that a combined therapy of vasopressin and epinephrine was superior to epinephrine monotherapy in enhancing the survival to hospital admission for post-cardiac arrest patients (0.76, 95%CI [0.64, 0.92]; *p* = 0.004) (Fig. 9).

### Vasopressin + methylprednisolone vs. epinephrine

#### Short-term survival/death at 28 or 30 days and neurologic outcomes

**Fig. 10:**
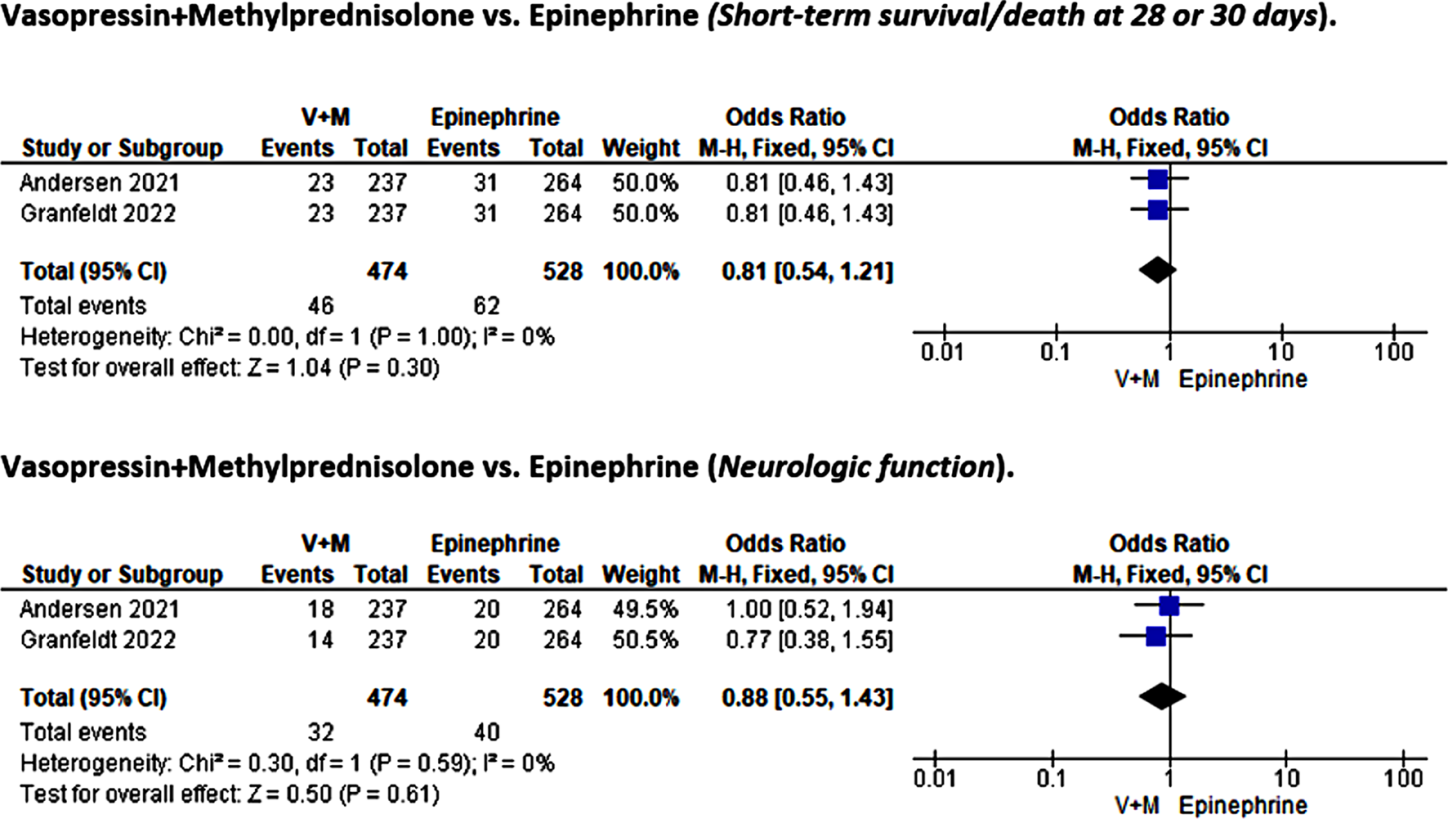
Forest plot comparison: vasopressin + methylprednisolone vs. epinephrine

Two studies involving 1,002 participants [24, 25] compared the effects of combinatorial therapy with vasopressin and methylprednisolone against epinephrine for short-term survival or death within 28 days and neurologic function outcomes. The heterogeneity test revealed no variation between the evaluated articles for both outcomes of interest (I²=0%, *p* = 1.00 and I²=0%, *p* = 0.59); therefore, a fixed-effects model was used in the meta-analysis. The pooled meta-analysis results revealed that vasopressin and methylprednisolone combined therapy was not effective in improving short-term survival outcomes (OR = 0.81, 95%CI [0.54, 1.21]; *p* = 0.30) or neurologic performance (OR = 0.88, 95%CI [0.55, 1.43]; *p* = 0.61) compared to epinephrine in post-cardiac arrest patients (Fig. 10).

### Vasopressin, steroids, and Epinephrine (VSE) vs. epinephrine alone

#### Survival to hospital discharge

**Fig. 11:**
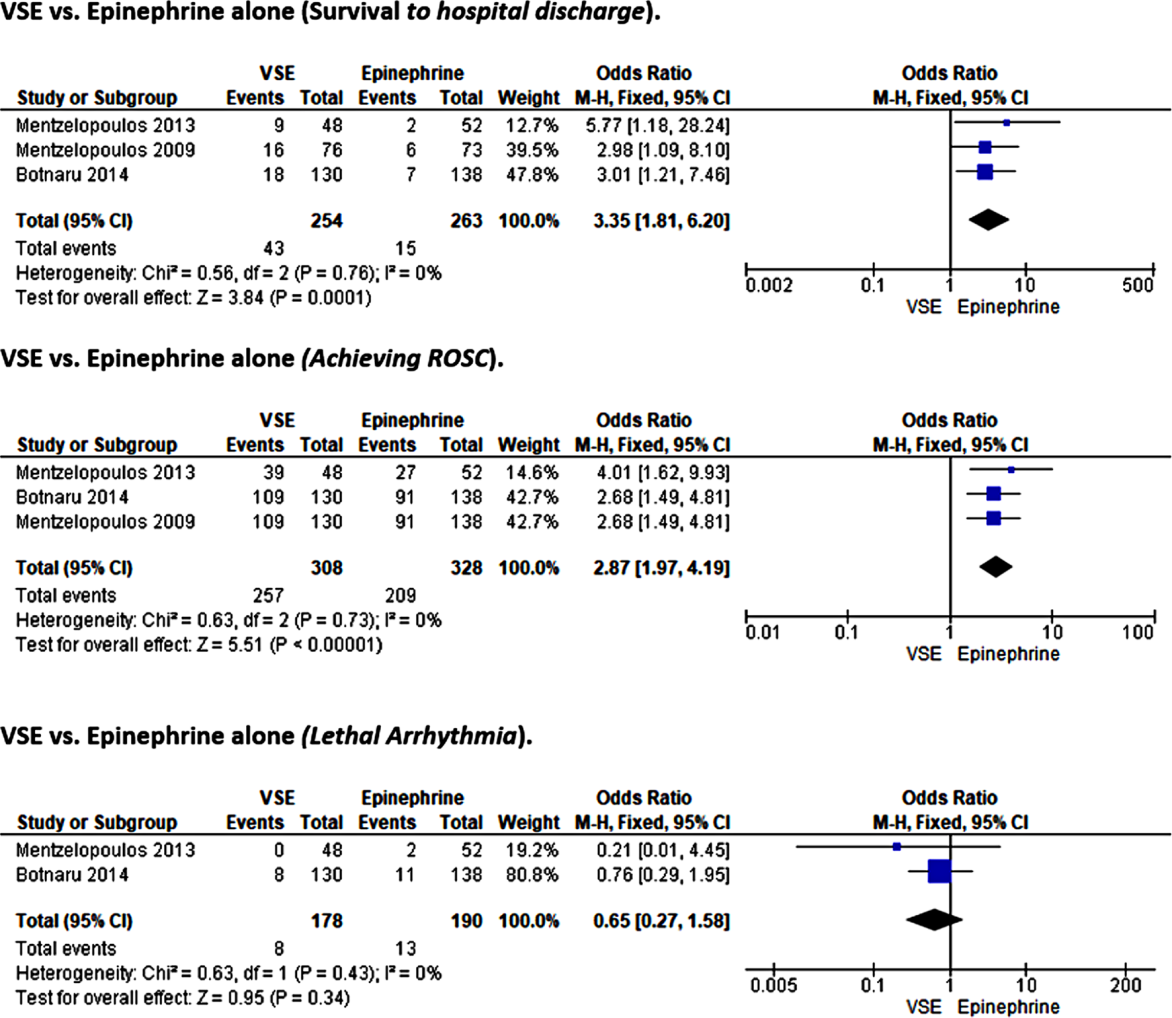
Forest plot of comparison: vasopressin, steroids, and epinephrine (vse) vs. epinephrine alone.

Three included studies, including 517 participants, reported survival to hospital discharge outcomes when comparing combined vasopressors (VSE) with epinephrine monotherapy [31–33]. There was no heterogeneity among these studies (I²=0%, *p* = 0.76), and a fixed effect model was used in the statistical analysis. The results showed significantly higher odds of survival to hospital discharge favoring Epinehrine alone (OR = 3.35, 95%CI [1.81, 6.2]; *p* < 0.001) over VSE therapy (Fig. 11).

#### ROSC

Three included studies, including 636 participants, reported ROSC outcomes when comparing combined vasopressors (VSE) with epinephrine monotherapy [31–33]; the heterogeneity test revealed no heterogeneity among the studies (I²=0%, *p* = 0.73), and Mantel–Haenszel’s fixed effect model was used in the meta-analysis. The pooled meta-analysis results showed significantly higher odds of achieving ROSC with Epinephrine alone (OR = 2.87, 95%CI [1.97, 4.19]; *p* < 0.001), indicating that combined therapy of VSE attributed no additional outcome benefits in post-cardiac arrest patients (Fig. 11).

#### Lethal arrhythmia

Two studies reported morbidity outcomes relating to lethal arrhythmia [31, 33]. With a sample population of 368 participants, the studies compared outcomes for patients in the combined vasopressor (VSE) arm against those in the Epinephrine arm. Since there was no inter-study heterogeneity (I²=0%, *p* = 0.43), the fixed effect model was used in the statistical pooling. Meta-analysis results showed a non-significant difference between the two treatment modalities on the risk of lethal arrhythmia (0.65, 95%CI [0.27, 1.58]; *p* = 0.34) (Fig. 11).

### Dopamine vs. norepinephrine

Among the included studies, only one study by De-Backer et al. compared the outcomes between dopamine and norepinephrine. Therefore, a meta-analysis of the outcomes was not possible. The study recognized dopamine and norepinephrine as first-line vasopressor agents for treating cardiac shock. In a multi-center randomized trial of 1,679 patients, the authors observed no significant difference between the treatment arms. Based on the outcomes, the 28-day death rate was 52.5% for the dopamine group and 48.5% for the norepinephrine group. However, the number of arrhythmic events was higher in the dopamine group than in the norepinephrine group (207 events [24.1%] vs. 102 events [12.4%], *p* < 0.001). Nonetheless, the study also indicated that dopamine was associated with increased death at 28 days compared with norepinephrine. Thus, the authors concluded that despite the non-statistically significant difference in death rates among the treatment arms, dopamine use was highly associated with more adverse events.

## Discussion

This systematic review and meta-analysis appraised the effectiveness and safety of vasopressors, the mainstay of resuscitation in cardiac arrest cases, regarding associated morbidity, mortality, and clinical outcomes in post-cardiac arrest patients to inform and support future evidence-based use of vasopressors in managing cardiac arrest. The clinical outcomes reported in this meta-analysis included differences in ROSC, survival-to-hospital admission rates, survival-to-hospital discharge, and improved neurologic performance. While mortality was reported through short-term survival or death within 28 days, morbidity was reported through myocardial infarction and lethal arrhythmia incidences. The search resulted in 30 high-quality studies (19 RCTs and 11 non-randomized cohort studies) with a large sample size of 459,708 participants.

Our findings indicate the superiority of epinephrine over placebo in terms of survival to hospital discharge and achieving ROSC but not in other short-term survival/ death at 28–30 days, survival to hospital admission, or neurological function outcomes. However, our analysis indicates a non-superiority of epinephrine over vasopressin in survival to hospital admission, ROSC, or neurological function outcomes. The differences in other outcomes, such as short-term survival/ death at 28–30 days or survival to hospital discharge with epinephrine versus vasopressin, could not be assessed due to a lack of data on these outcomes in the included studies. Similarly, no differences were observed in survival to hospital discharge or achieving ROSC with VE therapy or epinephrine monotherapy, although combined therapy significantly improved survival to hospital admission. Data was unavailable to compare other outcomes of interest with VE therapy or epinephrine monotherapy.

The included studies provided data to allow the assessment of only two outcomes of interest (short-term survival/death at 28 or 30 days and neurologic outcomes) with vasopressin-plus-methylprednisolone versus epinephrine; the treatment arm was non-superior over epinephrine for both outcomes. Further, the available data allowed the assessment of three outcomes of interest with combinatorial VSE therapy against epinephrine. While higher odds of survival to hospital discharge and ROSC favoring epinephrine over VSE therapy were observed, the risk of lethal arrhythmia was not statistically significant. Finally, only one study was identified in our search that compared the effects of dopamine and norepinephrine, and thus, a meta-analytic analysis could not be performed to compare the effects of the two treatment modalities. Nonetheless, the authors concluded that, despite the non-statistically significant difference in mortality rates between dopamine and norepinephrine treatment arms, dopamine use was highly associated with more adverse events.

## Limitations

The current study exhibited some crucial strengths. For instance, most of the included studies were RCTs with significantly larger sample sizes that were added to the quality and adjusted for some potential confounders. Two independent reviewers performed the search using defined search terms and strategies, thus limiting potential selection bias. Fundamentally, this study involved more peer-reviewed articles, making it more pervasive and reliable. However, the present review also has some limitations. First, the dose and drug sequences differed widely among the included studies. Another limitation was that most of the included articles were performed at single sites; thus, there is a possible chance of contamination bias in the experimental and control groups.

## Conclusions and future research insights

This study examines vasopressor use in cardiac arrest patients with ROSC, including in- and out-of-hospital cases. Findings, primarily based on adult patients, suggest epinephrine improves survival to hospital discharge and ROSC but not neurological outcomes or short-term survival. Combined vasopressor therapy shows no added benefits. Future research should include more high-quality RCTs and investigate epinephrine dosing and administration to understand organ perfusion and neurological outcomes better.

## Data Availability

The datasets used and/or analyzed during the current study are available from the corresponding author on reasonable request.
